# One-year follow-up of heart transplant recipient with cardiac rehabilitation: A case report

**DOI:** 10.1097/MD.0000000000019874

**Published:** 2020-04-24

**Authors:** Hee-Eun Choi, Chul Kim, Se-Heum Park

**Affiliations:** a Department of Rehabilitation Medicine, Inje University Haeundae Paik Hospital, Inje University College of Medicine, Busan; b Department of Rehabilitation Medicine, Inje University Sanggye Paik Hospital, Inje University College of Medicine, Seoul, Republic of Korea.

**Keywords:** cardiac rehabilitation, heart transplantation, oxygen consumption

## Abstract

**Introduction:**

Heart transplantation (HT) is known to be the final therapy for patients with advanced heart failure; however, the exercise capacity of these patients remains under the aged-predicted value after HT. Many studies have described the effectiveness and safety of cardiac rehabilitation (CR) in HT recipients. Nevertheless, long-term follow-up data of HT recipients undergoing CR are insufficient, and there is a lack of evidence on the long-term effects of CR. In this case report, we present the long-term benefits of CR in an HT recipient, including serial follow-up clinical data over 1 year.

**Patient concerns:**

A 48-year-old female patient underwent HT because of advanced dilated cardiomyopathy.

**Diagnosis:**

Cardiopulmonary exercise test showed reduced exercise capacity and pulmonary function. The grip power and quadriceps muscle strength were also decreased after HT.

**Interventions:**

The patient underwent a phase I CR program for 3 months, followed by a phase III CR program for 7 months. In the beginning, moderate-intensity continuous training was conducted. Thereafter, high-intensity interval training was implemented after a period of adjustment for interval training.

**Outcomes:**

The exercise capacity, 6-min walk distance, muscle strength, and vital capacity were improved after CR.

**Conclusion:**

CR in HT recipients may improve muscle strength and pulmonary function as well as exercise capacity, without serious cardiovascular complications. Phase III CR may help maintain exercise capacity in these patients.

## 1 Introduction

Cardiac rehabilitation (CR) is a comprehensive program including physician-prescribed exercise, education or counseling on cardiac risk factors, and psychosocial assessment.^[1]^ CR provides a multifaceted approach to enhance the exercise capacity, mental and social functioning, and prognosis of cardiac patients.^[2]^ For decades, exercise was restricted in heart transplantation (HT) recipients owing to the inappropriate heart rate (HR) response after heart denervation in these patients.^[3]^ However, progress in CR research has shown that aerobic exercise training may be effective in reversing the pathophysiological consequences associated with cardiac denervation and in preventing immunosuppression-induced adverse effects in HT recipients.^[3,4]^

Exercise capacity improves after HT when compared with that during end-stage heart failure; however, it continues to be subnormal when compared with the age-matched values in healthy individuals. Most studies show that the maximal oxygen uptake (VO_2max_) level in HT recipients range from 50% to 70% of that of the general population, and a reduced VO_2max_ level is generally associated with a poorer prognosis.^[3]^ As decreased exercise capacity affects the survival rate of HT recipients, like in other cardiovascular diseases, the importance of CR after HT is increasing.^[5]^

A recent systematic review on CR showed that long-term follow-up data are insufficient and there is a lack of evidence on the effects of CR on patient-related outcomes, except for exercise capacity, in HT recipients.^[6]^ In this case report, we present objective findings on the benefits of CR in an HT recipient, including serial longitudinal clinical follow-up data over 1 year.

## 2 Case report

A 48-year-old female patient was diagnosed with dilated cardiomyopathy and underwent insertion of an implantable cardioverter-defibrillator on May 26, 2017. However, she experienced progressive dyspnea with exercise intolerance, and her condition was classified as New York Heart Association class III. Thereby, she underwent HT on June 22, 2018.

When she first visited our CR clinic on July 27, 2018, 1 month after HT, she needed a wheel chair for long-distance mobility and had to stop for breath when walking at her own pace. As her health status did not allow conducting cardiopulmonary exercise testing, we prescribed comprehensive CR twice a day, 5 days a week, based on medical records and physical examination findings. During the first 2 weeks, aerobic exercise was performed using a bicycle. She also underwent training with lower-extremity strengthening exercises. After 2 weeks of comprehensive CR, she was able to walk on a treadmill independently.

The first CPET was performed at the second week of CR. The intensity of CPET was 1.6 metabolic equivalents (METs) initially and was manually increased by 0.3 METs every 3 minutes according to the manual protocol. After 12 minutes 30 seconds, the CPET was terminated according to the patient's request because of leg fatigue. At that time, the VO_2max_ was 12.3 mL/min/kg and the respiratory exchange ratio was 1.17. The maximal HR and myocardial oxygen demand were 100 bpm and 17,646 mmHg bpm, respectively. As the heart was denervated after HT, the HR recovery was delayed. HR increased up to 107 bpm in the recovery period for 4 minutes and then decreased. The 6-min walk test (6MWT) distance was measured to be 281 m (Table 1). The knee strength and hand-grip power were measured using an isokinetic dynamometer (BIODEX System 4 Pro^TM^; BIODEX, USA) and JAMAR Plus+ hand dynamometer (Performance Health, USA), respectively. Both knee extensor and flexor peak torques were < 30% of the age-predicted average values. The right and left hand-grip power was 64.3% and 67.0% of the age-predicted average value, respectively. A bioelectrical impedance analyzer (InBody S10; Biospace, Seoul, Korea) was used for the measurements of skeletal muscle mass (SMM) and phase angle (PhA) (Table 2). The parameters of pulmonary function were within the normal ranges; however, the respiratory muscle strength and peak cough flow (PCF) values were below the normal ranges (Table 3).

**Table 1:**
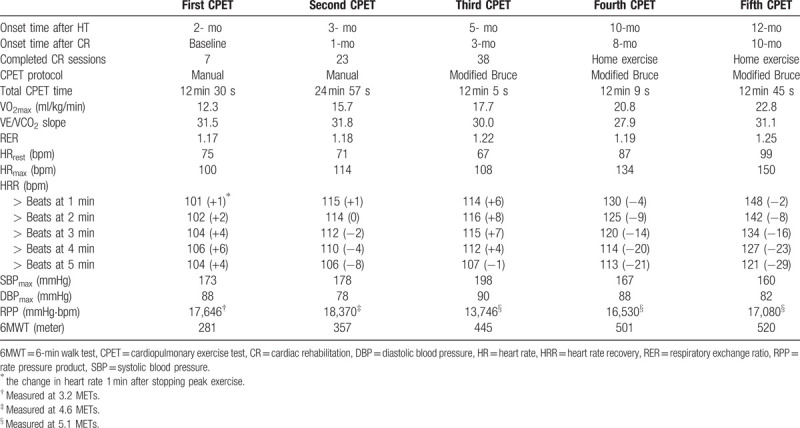
Results of cardiopulmonary exercise test.

**Table 2:**
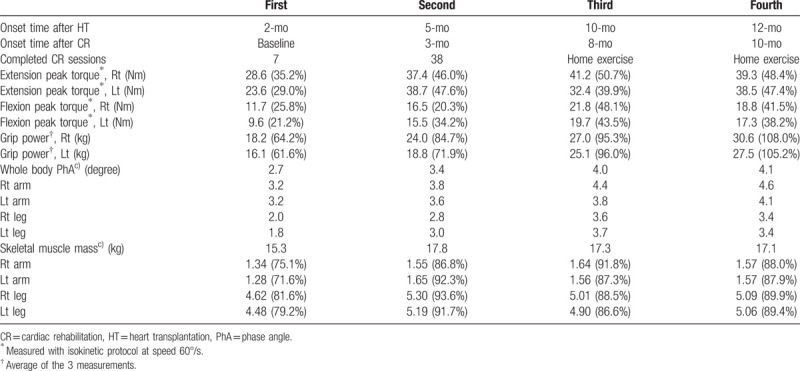
Muscle strength and mass.

**Table 3:**
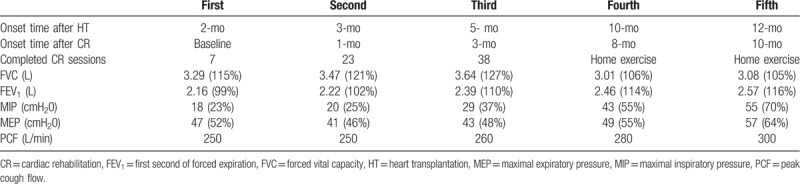
Results of pulmonary function test.

We prescribed the exercise program based on the first CPET and used a high-intensity interval training (HIIT) protocol with a treadmill. The patient exercised for a total of 50 minutes. Her program consisted of a 10-minute warm-up at 40% to 60% of the HR reserve, followed by four 4-minute intervals of walking on a treadmill at 85% of the HR reserve with three active pauses of 3-minute walking at 40% to 60% of the HR reserve and a 10-minute cool-down at 40% to 60% of the HR reserve. Adjustments were made to maintain the target HR at the equivalent percentages of the HR reserve. The MET values were calculated from the speed and slope of the treadmill, and were adjusted continuously to ensure that every training session was performed at the assigned HR. During exercise, the patient's blood pressure, HR, oxygen saturation, and electrocardiographic parameters were strictly monitored. Moreover, the exercises were supervised by an experienced physical therapist and a medical doctor who are specialized in CR.

At 1 and 3 months after starting CR, we conducted follow-up CPET and pulmonary function test. As the patient's exercise capacity and maximal HR were increased, we prescribed an increased exercise intensity based on the second CPET. She underwent HIIT with four 4-minute intervals of walking on a treadmill at 85% of the HR reserve (107–108 bpm) for 1 month. She experienced some complications that interfered with CR, such as varicella zoster infection; however, there were no serious cardiovascular events or complications. After she completed 38 sessions of CR, a third CPET was conducted with the modified Bruce protocol. As we changed the CPET protocol, the treadmill speed of the recovery phase was faster than that recommended in the manual protocol. Therefore, the HR recovery seemed delayed compared with that in the previous CPET.

After 38 sessions of CR, the patient's exercise capacity was 5.14 METs. She could finally walk and conduct most activities of daily living independently. Moreover, her respiratory muscle strength and PCF were also improved. Both knee strength and hand-grip power increased. The increase in SMM was 13% compared with that in the first bioelectrical impedance analysis. We recommended phase II CR; however, the patient refused outpatient CR because she lived far from the hospital that conducts CR. Therefore, we prescribed a home exercise program for phase III CR and educated the patient on how to perform the exercises.

**Figure 1:**
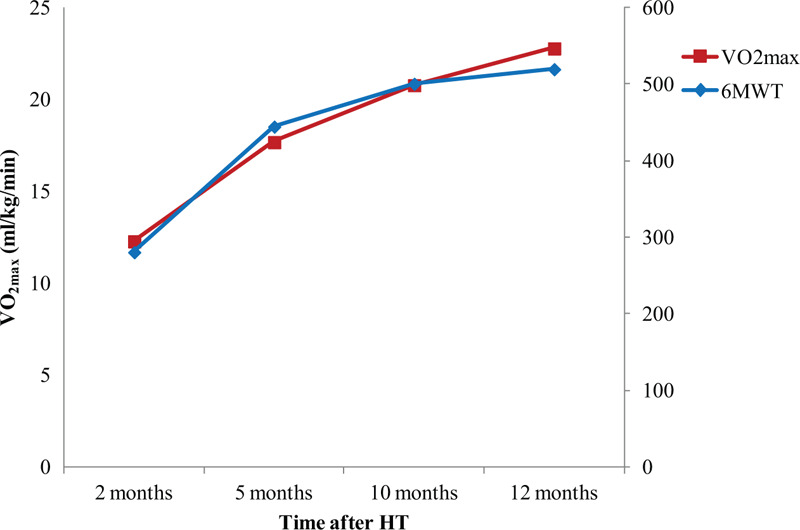
Serial follow-up of exercise capacity. 6MWT = 6-min walk test, HT = heart transplantation.

After 5 months of home exercise training, we conducted follow-up CPET at 10 months and 1 year after HT. Although the patient performed home exercises for 5 months, she achieved an exercise capacity of 6.51 METs and maximal HR of 150 bpm. The 6MWT distance was increased from 445 to 501 m (Fig. 1). Unlike in the previous CPET, the HR decreased immediately in the recovery period. This observation indicated that the pathophysiological consequences associated with cardiac denervation after HT had been reversed. Moreover, both grip power, the respiratory muscle strength, PCF and knee muscle strength were increased. However, the left knee extensor strength and SMM were slightly decreased.

**Figure 2:**
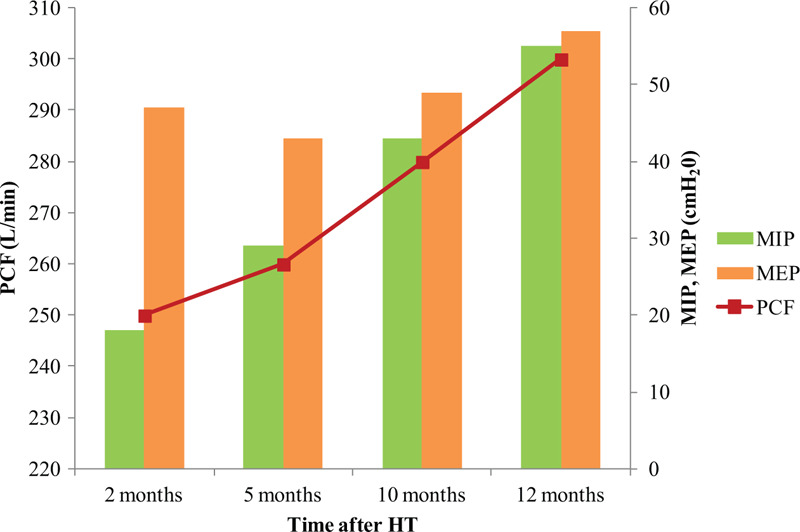
The improvement of respiratory muscle strength and peak cough flow. HT = heart transplantation, MEP = maximal expiratory pressure, MIP = maximal inspiratory pressure.

**Figure 3:**
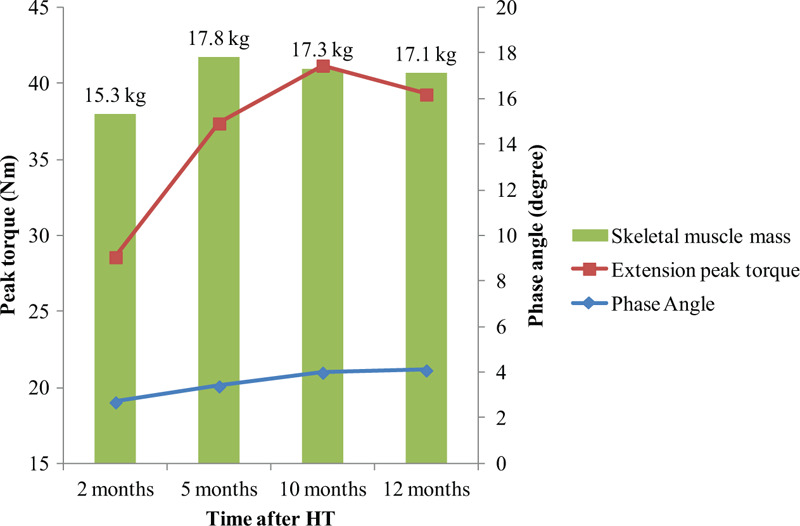
The changes of muscle strength, muscle mass and phase angle. HT = heart transplantation.

During 1 year of follow-up of the HT recipient, we observed that the oxygen consumption and maximal HR values constantly improved. However, her exercise capacity remained below the age-predicted value. At 1 year after HT, the HR recovery improved gradually. With respect to pulmonary function, although the forced vital capacity (FVC) and forced expiratory volume in 1 s (FEV_1_) were within the normal ranges at baseline, the respiratory muscle strength and PCF were very low before CR started. These parameters constantly increased until 1 year after HT (Fig. 2). The grip power gradually increased and reached the age-averaged value at the first year after HT; however, the lower-extremity muscle strength was below the 50% level of the age-averaged value. The SMM correspondingly increased to about 90% of the age-averaged value. The PhA showed constant increase during 1 year after HT (Fig. 3). With respect to the decrease in lower-extremity muscle strength during the home exercise period, it was believed to be related to lower-extremity pain. During the home exercise period, the patient periodically visited the outpatient department for evaluations. Her pain was concentrated on the hip joints, gluteus medius muscles, and tensor fascia latae, and she complained of gait disturbance. Although conservative treatment was provided, her hip pain persisted. Therefore, magnetic resonance imaging was conducted, which revealed avascular necrosis on both femoral heads due to post-HT steroid use. She underwent bilateral hip replacement arthroplasty at the hospital that conducted HT.

## 3 Discussion

To our knowledge, this is the first case report with time-series comparisons of maximal oxygen consumption, peak HR, pulmonary function, autonomic function, peripheral and respiratory muscle strength, and SMM in an HT recipient.

Studies on the effects and safety of CR in HT recipients have been increasing in recent years.^[7]^ Anderson et al systemically reviewed 10 randomized controlled trials of CR in HT recipients. They suggested that there is moderate-quality evidence showing that CR improves exercise capacity in HT recipients.^[6]^ The meta-analysis study of Didsbury et al reported that patients who underwent CR after HT achieved, on average, a 10.2% increase in VO_2max_ compared with recipients who did not.^[8]^ As reported in previous studies,^[4,6,8]^ this case showed a constant increase of VO_2max_, from 12.3 to 22.8 mL/min/kg, up to 1 year after HT. However, the change rate was 85.4%, which is much higher than that reported in other studies. According to Anderson et al's meta-analysis, most of the randomized controlled trials that performed CR in HT recipients started CR at least 1 year after HT. Only one study started CR within 2 weeks after HT. During 6 months of CR, the exercise capacity of patients increased from 9.2 to 13.6 mL/kg/min with a change rate of 49%.^[9]^ In the present case, although the duration of CR was only 3 months, the exercise capacity of the patient at 6 months after HT was higher than that in Kobashigawa report (17.7 vs 13.6 mL/min/kg).^[9]^ This difference in exercise capacity improvement seems to be associated with the baseline exercise capacity, exercise training intensity, HT surgical techniques, HT-related drugs, and post-HT management skills.

Unfortunately, studies with a follow-up of >1 year after HT are rare. Tegtbur et al reported that the exercise capacity at an average of 5.1 years after HT was improved in the CR group after 1 year of phase III CR compared with that in the control group.^[10]^ Kavanagh et al demonstrated that patients who underwent 16 months of outpatient CR, which started at 4.8 months after HT, had improved VO_2max_ from 22.2 ± 4 to 27.9 ± 6 mL/min/kg.^[11]^ However, by 12 years, the exercise capacity regressed to only slightly above the initial level (23.7 ± 6 mL/min/kg). In the present case, VO_2max_ was continuously increased over 1 year after HT. Moreover, the exercise capacity increased after 5 months of home exercise, although it remained under the age-predicted value.^[3]^ Therefore, long-term CR may be needed, with gradual progression from phase I to phase III CR.

HT recipients show decreased maximum HR and delayed chronotropic response because of the denervated heart. Such inappropriate physiologic responses are associated with decreased exercise capacity in these patients.^[6]^ The effectiveness of CR in improving the chronotropic response has not been well established in HT recipients, and few studies have demonstrated the recovery of chronotropic response after CR.^[2,12]^ Dall et al compared patients who underwent HIIT with those who underwent continued moderate exercise training, and observed improvement of HR recovery in both groups after 12 weeks of training.^[2]^ Karapolat et al compared a hospital-based CR group with a home-based CR group, and found significant improvement of the HR reserve in the hospital-based CR group after 8 weeks of training.^[12]^ Further, there was no significant change in HR recovery at 1 minute after peak exercise in both groups.^[12]^ Consistent with these findings, the present patient showed substantially increased HR reserve up to 1 year after HT; however, the HR recovery was insufficient after 3 months of CR. We also found that the chronotropic incompetence was continuously improved during the long-term CR period.

Muscle atrophy caused by postoperative restrictions and adverse effects of immunosuppressive agents is commonly found in HT recipients, leading to poor functional capacity after HT.^[13]^ Haykowsky et al applied 12 weeks of CR in HT recipients, and achieved improvements not only in the maximal strength in leg- and chest-press, but also in lean tissue mass in the legs.^[4]^ Kavanagh et al found that 16 months of exercise training was associated with a 2-kg increase in lean body mass.^[11]^ Braith et al reported that 6 months of aerobic and strength training increased the lean body mass,^[14]^ chest-press strength,^[14,15]^ and leg-extension strength.^[14,15]^ These investigators also found significant increases in vastus lateralis type 1 (oxidative) myosin heavy chain isoform, citrate synthase, and lactate dehydrogenase enzymes.^[15]^ Consistent with these results, we found that 12 weeks of hospital-based CR increased the knee extension/flexion peak torque, grip power, and SMM. However, the overall muscle strength and the muscle mass of the extremities were not increased in the home-based CR period. These findings were presumed to be due to the patient's hip pain during home exercise training. In addition, there is a possibility that home-based CR might not have been properly performed compared with hospital-based CR. Interestingly, we found that PhA, which represents cellular health and is a parameter used in recent studies to evaluate the recovery and prognosis of critically ill patients,^[16]^ showed a constant increase during long-term CR. Although there has been no study on PhA in HT recipients, PhA may be used as an indicator of general health or functional status in HT recipients.

Meanwhile, pulmonary function before HT is related to chronic heart failure and manifests as restrictive ventilatory abnormalities. Moreover, it is known that FEV_1_ and FVC recover after HT.^[17]^ Our patient showed normal ranges of FEV_1_ and FVC, similar to patients in previous studies. However, the maximal inspiratory pressure, maximal expiratory pressure, and PCF, which represent respiratory muscle strength, were below the normal predicted values. During CR in this case, the maximal inspiratory pressure, maximal expiratory pressure, and PCF continuously improved. Aerobic exercise is known to improve pulmonary function by strengthening the respiratory muscles, improving thoracic mobility, and enhancing the balance between lung and chest elasticity.^[18]^ These findings suggest that not only skeletal muscle strength but also respiratory muscle strength can decrease after HT.

This case showed that CR programs can be successfully performed in HT recipients, with objective improvements in exercise capacity, chronotropic responses, skeletal and respiratory muscle strength, and SMM. Concerning safety, there were no life-threatening events or complications during long-term CR, even with the HIIT protocol, in the present case. However, it is difficult to conclude that HIIT is sufficiently safe for patients with HT. Therefore, large-scale multicenter randomized studies are needed to confirm the long-term efficacy and safety of CR in the future.

## Author contributions

**Conceptualization:** Hee-Eun Choi.

**Investigation:** Hee-Eun Choi, Se-Heum Park.

**Resources:** Hee-Eun Choi.

**Writing – original draft:** Hee-Eun Choi, Chul Kim, Se-Heum Park.

**Writing – review & editing:** Hee-Eun Choi, Chul Kim.

Se-Heum Park orcid: 0000-0002-0349-4865.

## References

[1] Dalal HM Doherty P Taylor RS Cardiac rehabilitation. BMJ 2015;351:h5000. 26419744 10.1136/bmj.h5000 PMC4586722

[2] Dall CH Snoer M Christensen S Effect of high-intensity training versus moderate training on peak oxygen uptake and chronotropic response in heart transplant recipients: a randomized crossover trial. Am J Transplant 2014;14:2391–9. 25135383 10.1111/ajt.12873

[3] Nytrøen K Gullestad L Exercise after heart transplantation: an overview. World J Transplant 2013;3:78–90. 24392312 10.5500/wjt.v3.i4.78 PMC3879527

[4] Haykowsky M Taylor D Kim D Exercise training improves aerobic capacity and skeletal muscle function in heart transplant recipients. Am J Transplant 2009;9:734–9. 19344465 10.1111/j.1600-6143.2008.02531.x

[5] Yardley M Gullestad L Bendz B Long-term effects of high-intensity interval training in heart transplant recipients: A 5-year follow-up study of a randomized controlled trial. Clin Transplant 2017;31:e12868. 10.1111/ctr.12868 27865004

[6] Anderson L Nguyen TT Dall CH Exercise-based cardiac rehabilitation in heart transplant recipients. Cochrane Database Syst Rev 2017;4:CD012264. 28375548 10.1002/14651858.CD012264.pub2 PMC6478176

[7] Wenger NK Current status of cardiac rehabilitation. J Am Coll Cardiol 2008;51:1619–31. 18436113 10.1016/j.jacc.2008.01.030

[8] Didsbury M McGee RG Tong A Exercise training in solid organ transplant recipients: a systematic review and meta-analysis. Transplantation 2013;95:679–87. 23364480 10.1097/TP.0b013e31827a3d3e

[9] Kobashigawa JA Leaf DA Lee N A controlled trial of exercise rehabilitation after heart transplantation. N Engl J Med 1999;340:272–7. 9920951 10.1056/NEJM199901283400404

[10] Tegtbur U Busse M Jung K Phase III Rehabilitation nach Herztransplantation. Zeitschrift für Kardiologie 2003;92:908–15. 14634760 10.1007/s00392-003-0968-6

[11] Kavanagh T Mertens DJ Shephard RJ Long-term cardiorespiratory results of exercise training following cardiac transplantation. Am J Cardiol 2003;91:190–4. 12521633 10.1016/s0002-9149(02)03108-9

[12] Karapolat H Eyigor S Zoghi M Effects of cardiac rehabilitation program on exercise capacity and chronotropic variables in patients with orthotopic heart transplant. Clin Res Cardiol 2008;97:449–56. 18317667 10.1007/s00392-008-0648-7

[13] Stewart KJ Badenhop D Brubaker PH Cardiac rehabilitation following percutaneous revascularization, heart transplant, heart valve surgery, and for chronic heart failure. Chest 2003;123:2104–11. 12796195 10.1378/chest.123.6.2104

[14] Braith RW Limacher MC Leggett SH Skeletal muscle strength in heart transplant recipients. J Heart Lung Transplant 1993;12 (6 Pt 1):1018–23. 8312302

[15] Braith RW Magyari PM Pierce GL Effect of resistance exercise on skeletal muscle myopathy in heart transplant recipients. Am J Cardiol 2005;95:1192–8. 15877992 10.1016/j.amjcard.2005.01.048

[16] Gunn SM Halbert JA Giles LC Bioelectrical phase angle values in a clinical sample of ambulatory rehabilitation patients. Dyn Med 2008;7:14. 18782456 10.1186/1476-5918-7-14 PMC2551587

[17] Ravenscraft SA Gross CR Kubo SH Pulmonary function after successful heart transplantation. One year follow-up. Chest 1993;103:54–8. 8417937 10.1378/chest.103.1.54

[18] Angane E Navare A Effects of aerobic exercise on pulmonary function tests in healthy adults. Int J Res Med Sci 2017;4:2059–63.

